# Traveling Letter, No. X

**Published:** 1844-12

**Authors:** 


### TRAVELING LETTERS FROM THE SENIOR EDITOR.

NO. X.

UPPER MISSISSIPPI, October 11, 1844.

*To my Colleagues:*

GENTLEMEN:—It is now the third day since I began to descend this noble river, of which I must give you a passing notice. I have the distinction of being its last voyager, up to this time, as Father Marquette 170 years ago was its first—Indians excepted. With all my predilections for our cherished Ohio, I am bound to admit, that the Upper Mississippi is more beautiful. Its banks, and bottoms, and hills, present greater variety in form and elevation; and the alternation of prairie and woodland affords a more agreeable diversity. The stream itself is not very serpentine, and although now greatly reduced in depth, it fills its bed, and few rocks or sand-bars are to be seen. The number of snags and sawyers is still fewer than in the Ohio, for its banks have less density of forest, and its sands are not deep enough to catch and fix the floating trees. In all these respects, it is incomparably superior to the Missouri. Its depth and current appear to be tolerably uniform, except at the two rapids, where the former is shoal and the latter swift, for several miles. More than either the Missouri or Ohio, the Mississippi has a disposition to divide into channels and form islands, four of which currents may sometimes be seen at one place, and one or more islands or islets are always in view. They are not mere unstable beds of sand, like those of the Missouri, but permanent, and decked out with shrubs and trees to the waters edge; nor are they defaced by the great deposits of drift wood, which, disfigure the islands below the mouth of the Missouri, and cause an unusual growth up stream, while the lower ends are washed away by the current. It is indeed to its 'thousand isles' fringed with willow, that this part of the great river owes one of its pleasantest charms. In color, the Missouri, Upper Mississippi, and Ohio, has each its peculiarity. The first, even when low, displays a yellowish sand—turbidness, which it imparts to the waters of both the others, after its union with them; so that the main trunk below the latitude of 39° is never transparent. The Upper Mississippi, free from transported materials, presents a dark or brownish tint, which is perceptible, when dipped up, but does not destroy its clearness. Two years ago, I saw this die, still deeper, in some of the streams of Michigan and northern Ohio; where it was ascribed to the tanno-gallate of iron, formed in the swamps from which they were said to originate. The pellucid waters of the Ohio, when low, have a dull green or yellow tinge, quite distinguishable from that of the Missisisppi, before its defilement by the muddy Missouri. By the way, it is to this polluting power of that river, that it owes one of its claims to be regarded as the greater of the two, while another rests upon its driftwood. The matters which it brings down, do, indeed, give to the united river, the same character with the Missouri above, but these imperfections are from establishing its pre-eminence over the pure and magnificent stream, which it deforms. But not to make myself too violent a partisan in this *bellum fluvium*, I will only add, that water-fowl, because they can see to fish in it, are much more numerous along the Mississippi than the Missouri; and that the cotton-tree, on the contrary, is more abundant, attains to greater elevation, and extends to a higher altitude, on the latter than the former.

The Upper Mississippi participated but sparingly in the great flood of the past summer. When crossing Black river in Wisconsin, the largest tributary below the river of that name, I found that it had not been swollen to any extraordinary height. The streams below its mouth might have been, but are of much less magnitude than those which contribute to the Missouri, in corresponding latitudes. On the whole then, we may conclude, that the unprecedented rains of April, May, and June, did not extend further north than the latitude of 42° 30′ or 42°. Had they fallen in equal proportion to the sources of the Mississippi and Missouri, and on the Ohio, the inundation would have been still more overwhelming.

St. Louis, Oct. 12, 1844.

To pass from geography to pharmacy, I must tell you something about the manufacture of Castor Oil in this city and some of the neighboring parts of Illinois. It seems to have been commenced by Mr. Adams, of Edwardsville, (*Peck's* Gazetteer,) about 20 years ago. The produce of the first year was 500 gallons, which he was then enabled to sell at two dollars a gallon. In 1831, he made 10,000 gallons, which he sold at 75 cents. From a gentleman of this city, who owns a steam oil press, I have some additional statistics of this important manufacture.

According to his statement, the quantity now manufactured in Illinois and Missouri, is above 100,000 gallons a year; which is the produce of from 8 to 10,000 acres of land. The crop varies from 10 to 15 bushels an acre, each bushel yielding about 2 gallons of oil. Its price ranges from 75 cents to $1 a gallon. In these latitudes the crop is somewhat uncertain, being liable to injury from long-continued droughts and early frosts. Indiana, Virginia, and New Jersey furnish, together, from 25 to 30,000 gallons a year: Tennessee as much, perhaps, as she consumes. The castor oil bean flourishes well in Mexico and the West Indies; but the importation of oil is greatly diminished from what it once was; at the same time the price is much reduced, and the quality greatly improved.

Ohio River, October 16, 1844.

This day will finish my *rambles* of nearly seven months, in which, by land and water, I have traveled about 6,200 miles; and still left large portions of this boundless and beloved West of ours unvisited. I am now on my 29th steamboat, not one of which has been overtaken by any casuality. Indeed, I have come to believe it is safer, in this country, to travel by water than land. All the stage coaches and diligences of Europe and the United States could not, in the same time, transport the number of persons conveyed from place to place, by the boats on the Lakes, the Gulf, and the Mississippi with its tributaries, and still, how few accidents, necessarily involving the lives of passengers, do we hear of! But I have also traveled in skiffs, on rail roads, in stages, buggies, common wagons, on horse back, mule back, and on foot; by night and by day, without arms, and without having suffered a single aggression, robbery, or theft; which certainly speaks well of the state of society, though, it must be confessed, that our country is not without ruffians and robbers, to the production of which our steamboats undoubtedly contribute their full share, by the drinking, gambling and licentious conversation of too many of their passengers. Still, these sources of corruption to clerks, mates, pilots, engineers, cabin boys, and young passengers, are gradually drying up. Table drinking is almost at end, and those who go to bars, are fewer and shyer than in days of yore. And here I cannot help asking, why the General Government, which professes to legislate in various ways, for the safety of passengers, and requires every boat to be registered and licensed, does not prohibit the sale of intoxicating drinks on the whole, and forbid their use to those who manage them, except while in port? Such a prohibition, rigidly carried out, would soon throw the control of them into the hands of men who practise total abstinence; and contribute at least as much to their safety, as the thousands which are expended on the removal of snags and sawyers. That our members of Congress do not move in this matter, seems strange, indeed, and should, perhaps, be ascribed to their desire not to be without an occasional 'cock-tail,' when on their way to Washington, after the immense amount of drinking which their getting into office has required. If intoxicating drinks were banished from our boats, those who manage and those who travel on them, might resort to what is far better for health, both of mind and body,—an occasional cup of strong, hot coffee with a cracker, which could be sold at the *Cafe* of the boat, for half the price of the glass of poison, and still afford a good profit to the proprietors.

In conclusion I must say, that from all the facts I have been able to collect, steamboat traveling is healthful, especially in reference to autumnal fever. Our boats' crews probably average 30 persons each, and in general they are more exempt from intermittent and remittent fevers, than the people who reside on the banks of the rivers which they navigate. The same appeared to me to be the case on the Ohio and Erie canal, when I traveled upon it in 1842.

I shall now, gentlemen, bring this gossipping correspondence to a close. If I have figured rather conspicuously in it myself, you must recollect the traveler's license; if I have crowded out your own valuable lucubrations, they will only flow on our readers more copiously in future, and be even all the purer, from having had time to deposit all extraneous mixtures; as the dammed-up stream throws down its sediment, and then flows on in a deep transparent current when the obstacle is removed; finally, if those for whom we write, are rejoiced, that No. X. is the last, so am I, and this common sympathy, will I hope, make us part as friends.

Your very obedient servant,

